# Opioid prescriptions at the point of surgery, bone metastasis, or death among patients with breast cancer in Japanese acute care hospitals: a claims-based, retrospective, longitudinal study

**DOI:** 10.1007/s00520-023-07805-4

**Published:** 2023-06-02

**Authors:** Manami Yoshida, Kosuke Iwasaki, Mitsunori Miyashita, Toshiaki Saeki, Yasuhide Morioka, Shinzo Hiroi, Eiko Shimizu

**Affiliations:** 1 Medical Affairs, Shionogi & Co., Ltd., Tokyo, Japan; 2 Social Cooperation Program of IT Healthcare, Graduate School of Pharmaceutical Sciences, The University of Tokyo, Tokyo, Japan; 3 Milliman, Inc., Tokyo, Japan; 4 Department of Palliative Nursing, Health Sciences, Tohoku University Graduate School of Medicine, Miyagi, Japan; 5 Department of Breast Oncology, Saitama Medical University International Medical Center, Saitama, Japan

**Keywords:** Breast neoplasm, Japan, Longitudinal study, Opioid, Pain management, Real world data

## Abstract

**Purpose:**

Breast cancer is the most common cancer among Japanese women and often yields a better prognosis than other cancers. However, few studies have been conducted on pain control using opioids in Japan. In this study, we aimed to examine actual opioid use among breast cancer patients.

**Methods:**

Breast cancer patients were defined as female patients with a first breast cancer diagnosis during the observational period in an acute care hospital database (April 2008 − February 2020). We examined the percentage of patients prescribed opioids, the opioid amount per patient, and the opioid dosage per day around surgery, bone metastasis diagnosis, or death.

**Results:**

Overall, 217,722 breast cancer patients were identified. The percentage of patients prescribed opioids and the average amount of opioids per patient were highest in the month of surgery, 78% and 27 morphine milligram equivalents (MMEs), respectively. The average opioid dosage increased with time after surgery from 19 to 28 MMEs. Around bone metastasis, the percentage of patients prescribed opioids and the average opioid amount per patient peaked one month after the diagnosis, 31% and 371 MMEs, respectively. The average opioid dosage gradually increased from 22 to 35 MMEs in succeeding days after a bone metastasis diagnosis. The percentage of patients prescribed opioids and the average opioid amount per patient increased as the month of death approached.

**Conclusion:**

We investigated opioid prescription trends around clinical events in breast cancer patients on a large scale in Japan. These results may be useful to control cancer pain among breast cancer patients.

**Supplementary information:**

The online version contains supplementary material available at 10.1007/s00520-023-07805-4.

## Introduction

According to the 2019 Cancer Statistics provided by the National Cancer Center, breast cancer is the most common cancer among women in Japan [1]. The 10-year overall survival rate for patients with breast cancer is reportedly over 80% [2, 3]. Patients with breast cancer often experience events that cause pain over a long period, such as surgery or bone metastasis [4]. Opioids are normally used to control pain [5]. Consequently, pain control with the appropriate use of opioids is considered to have significant positive impacts in patients with this disease.

In an international study, approximately 50% of breast cancer survivors reported pain lasting over several months after breast cancer surgery [6–9]. After bone metastasis occurs, more than 80% of patients experience bone pain [10], and furthermore, the percentage of patients who experience pain three months before their death is reported to be 75.8% [11].

In the USA, 35.3% of patients with breast cancer were prescribed opioids within a year after the diagnosis of breast cancer from 2013 to 2017 [12]. A total of 34.2% of patients were prescribed opioids in 2018 [13]. However, the pain of patients with breast cancer was not adequately relieved [14].

In other countries, such as the USA, opioids were broadly prescribed; otherwise, in Japan, opioids were strictly under control to prevent casual long-term prescription of opioids, which may cause opioid abuse and addiction [15]. In Japan, some investigations of actual opioid prescriptions in patients with cancer, including breast cancer, have already been performed. One study reported the proportion of analgesic prescriptions, including nonsteroidal anti-inflammatory drug (NSAIDs) prescriptions, acetaminophen, and opioids, by type of drug and cancer site, and prescription status according to treatment phase was analyzed for patients with five major cancers without subanalysis by site [16]. In that study, the average proportion of opioid prescriptions per month was 4.2% in patients with breast cancer. Additionally, an average percent of opioid prescription per month after the surgery was reported as 6.7% in any cancer included in the analysis. Another study in Japan investigated opioid prescription status in patients with cancer, including breast cancer, using real-world data [17]. However, it is debatable whether the recent data on pain control among patients with breast cancer in Japan are sufficient to yield useful inferences since the prescription status by treatment phase or in the overall disease course has not been reported.

We investigated opioid prescription status around clinical events in patients with breast cancer using a large-scale database containing data from DPC hospitals. DPC hospitals are acute care hospitals that use the Japanese Diagnosis Procedure Combination/Per-Diem Payment System (DPC/PDPS) [18]. There were 1757 DPC hospitals with approximately 480,000 beds, representing approximately 89% of all acute care beds in Japan as of April 1, 2020 [19]. In this research, we focused on pain at the end of life, after the diagnosis of bone metastasis and after surgery. The first two of three is known as representative pain in cancer [20], and the last is that some research reports the long-term use of opioids after surgery [21].

## Methods

### Study design and data source

A claims-based retrospective study was performed using the DPC hospital database from April 2008 to February 2020, provided by Medical Data Vision Co., Ltd. (Tokyo, Japan). The database included data from 449 DPC hospitals, which represent 26% of all Japanese acute care hospitals and serve approximately 367 million patients in total. The database contained health insurance claims data, including diagnosis, medical procedure, and prescription data for both inpatients and outpatients, as well as discharge summary data for inpatients associated with the DPC/PDPS.

This study was approved by the Ethics Committee of the University of Tokyo (No 31–32). All procedures were in accordance with the Ethical Guidelines for Medical and Health Research Involving Human Subjects by the Ministry of Education, Culture, Sports, Science, and Technology and the Ministry of Health, Labor, and Welfare, Japan. Informed consent was waived because the collected data were anonymized for secondary use.

### Patient identification

We defined patients with breast cancer as female patients who had a definitive diagnosis of breast cancer and first diagnosis of breast cancer during the observational period. Breast cancer diagnosis was defined as the presence of one of the first three codes of C50 in the International Classification of Diseases, 10th Revision (ICD-10). The first diagnosis was defined as the “FromDate” in the database, which was recorded at the first diagnosis of each disease, regardless of the observational period. The observational period was the duration from the first to the last record of medical practice for each patient in the database. It should be noted that patients with breast cancer included in this study could have multiple cancers. We did not exclude these patients to reflect the actual situation of opioid prescriptions for patients with breast cancer.

### Analysis

We performed descriptive statistical analysis of opioid prescription status indexed by surgery, bone metastasis, and death among the identified breast cancer patients. Opioids were defined as any drug with a generic name listed in SI Table 1, including both strong and weak opioids. Opioids prescribed for rescue therapy (sublingual tablets and buccal tablets containing fentanyl citric acid) were excluded from all analyses. Dosages of opioids were converted to morphine milligram equivalents (MMEs) by referencing guidelines [10, 22, 23] and an article [24]. Surgery related to breast cancer was defined based on the name of the medical procedure (SI Table 2). Bone metastasis was defined as diagnosis with any of the following standard disease names: “bone metastasis in cancer,” “metastatic bone tumor,” or “breast cancer bone metastasis.” Death was defined as an outcome of death at discharge as recorded in the discharge summary data. We defined breast cancer–related deaths as deaths associated with the C50 ICD-10 code, recorded as any main condition, trigger-for-hospitalization condition, or greatest-resource consuming condition. Patients who experienced each index event were considered the target patients for each analysis.

## Results

### Patients

A total of 217,722 breast cancer patients were identified in the database (see the flow diagram in SI Fig. 1). Among the patients observed in 2019 (138,815 patients, 1,217,435 patient-months), the average age was 64.3 years, and the number of patients who were prescribed opioids at least once in 2019 was 26,768 (19.3%). Among the 1,217,435 patient-months, opioids were prescribed for 42,490 patient-months (3.5%).

**Fig. 1:**
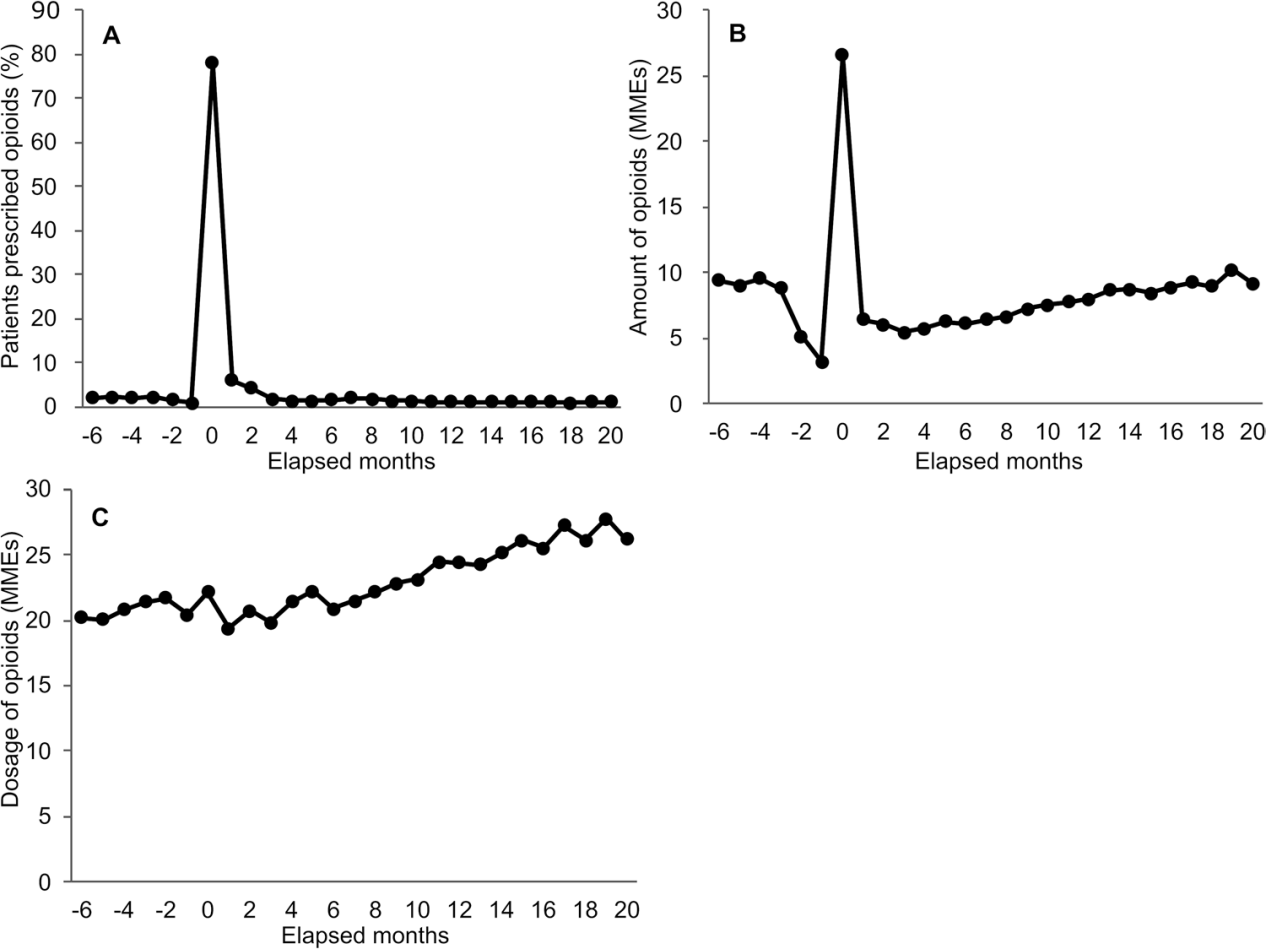
Opioid prescription status by elapsed months from the first surgery related to breast cancer. Target patients are those who underwent surgery related to breast cancer.** A** Percentage of patients prescribed opioids among target patients. **B** Average amount of opioids per target patient. **C** Average opioid dosage per day. The amount and dosage of opioids are presented in morphine milligram equivalents (MMEs). MMEs, morphine milligram equivalents

### Opioid prescription status with first surgery related to breast cancer

The number of target patients undergoing surgery was 110,211 (Table 1). The average (standard deviation [SD]) period from first diagnosis to surgery was 2.0 (4.0) months. More than 2% of patients had opioid prescriptions 3 to 6 months before surgery; this percentage decreased to 1.0% 1 month before surgery and then sharply increased from less than 2 to 78% in the month that the surgery was performed. The percentage subsequently decreased to approximately 1 − 2% at three months after surgery (Fig. 1A and SI Table 3). The number of MMEs per surgical patient was more than 8 as of three to six months before surgery; this number decreased to approximately 3 at one month before surgery and then increased to 26 in the month when the surgery was performed (Fig. 1B and SI Table 3). The percentage of patients who were prescribed opioids and the number of MMEs per surgical patient showed similar trends. On the other hand, the number of days’ supply per patient decreased dramatically from approximately 15 days in the month before surgery to approximately 2 days in the month surgery was performed; then, it recovered to the preoperative baseline (SI Table 3). The opioid dose per patient per day was roughly consistent until 10 months after surgery, after which it gradually increased until 19 months after surgery (Fig. 1C and SI Table 3).

**Table 1:** The numbers of target patients undergoing surgery and those prescribed opioids by months from surgery

Elapsed months	Patients^a^, No	Patients^a^ prescribed opioids, No
− 6	10,614	229
− 5	11,940	268
− 4	13,473	296
− 3	17,536	400
− 2	38,523	606
− 1	87,320	892
0	110,211	85,926
1	106,296	6528
2	101,850	4337
3	98,695	1874
4	96,074	1442
5	93,822	1343
6	91,907	1514
7	89,547	1895
8	87,605	1610
9	85,804	1274
10	84,041	1178
11	82,513	1070
12	80,832	1084
13	77,815	995
14	75,723	923
15	73,894	876
16	72,140	842
17	70,629	827
18	69,183	760
19	67,225	781
20	65,682	749

*No*, Number. ^a^Patients with breast cancer who underwent surgery

### Opioid prescription status with first bone metastasis diagnosis

**Fig. 2:**
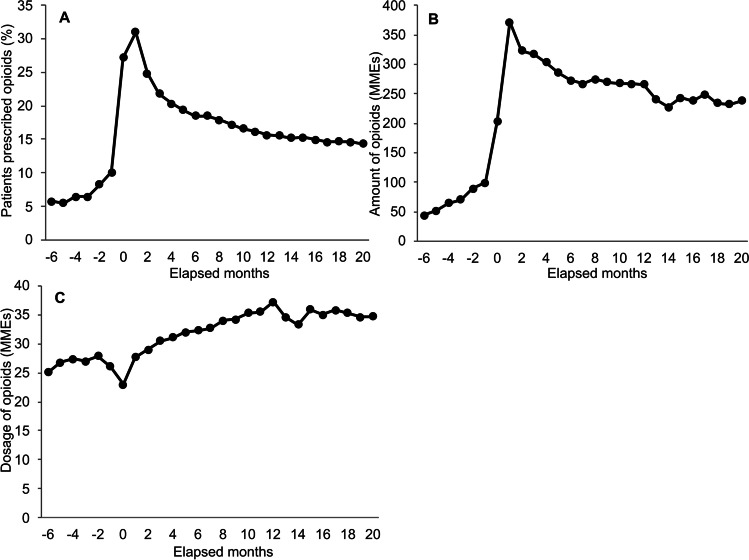
Opioid prescription status by elapsed months from first bone metastasis. Target patients were those who were diagnosed with bone metastasis after breast cancer diagnosis. **A** Percentage of patients prescribed opioids among target patients. **B** Average amount of opioids per target patient. **C** Average opioid dosage per day. The amount and dosage of opioids are presented in morphine milligram equivalents (MMEs). MMEs, morphine milligram equivalents

The number of target patients diagnosed with bone metastasis was 13,146 (Table 2). The average (SD) period from first diagnosis to bone metastasis was 8.8 (16.4) months. The percentage of patients who were prescribed opioids was higher after the first bone metastasis diagnosis than before diagnosis, and it was 31% 1 month after the diagnosis (Fig. 2A and SI Table 4). The number of MMEs per patient diagnosed with bone metastasis was also higher after the diagnosis of bone metastasis than before diagnosis (Fig. 2B and SI Table 4). After bone metastasis was diagnosed, the percentage of patients prescribed opioids and the amount of MMEs per patient diagnosed with bone metastasis decreased in a similar manner, whereas the opioid dosage per patient per day gradually increased from approximately 22 MMEs in the month bone metastasis was diagnosed to 37 MMEs 12 months after bone metastasis was diagnosed (Fig. 2C and SI Table 4).

**Table 2:** The number of target patients diagnosed with bone metastasis and those prescribed opioids by months from first bone metastasis diagnosis

Elapsed months	Patients^a^, No	Patients^a^ prescribed opioids, No
− 6	4085	235
− 5	4231	232
− 4	4396	285
− 3	4598	297
− 2	4975	413
− 1	6165	623
0	13,146	3568
1	11,301	3505
2	9903	2464
3	9111	1983
4	8550	1738
5	8085	1566
6	7671	1420
7	7305	1354
8	6946	1246
9	6642	1143
10	6336	1053
11	6091	986
12	5821	909
13	5541	861
14	5309	806
15	5111	784
16	4898	732
17	4676	684
18	4521	663
19	4311	631
20	4130	592

*No*, Number. ^a^Patients with breast cancer who were diagnosed with bone metastasis

#### Opioid prescription status at the time of death due to breast cancer

**Fig. 3:**
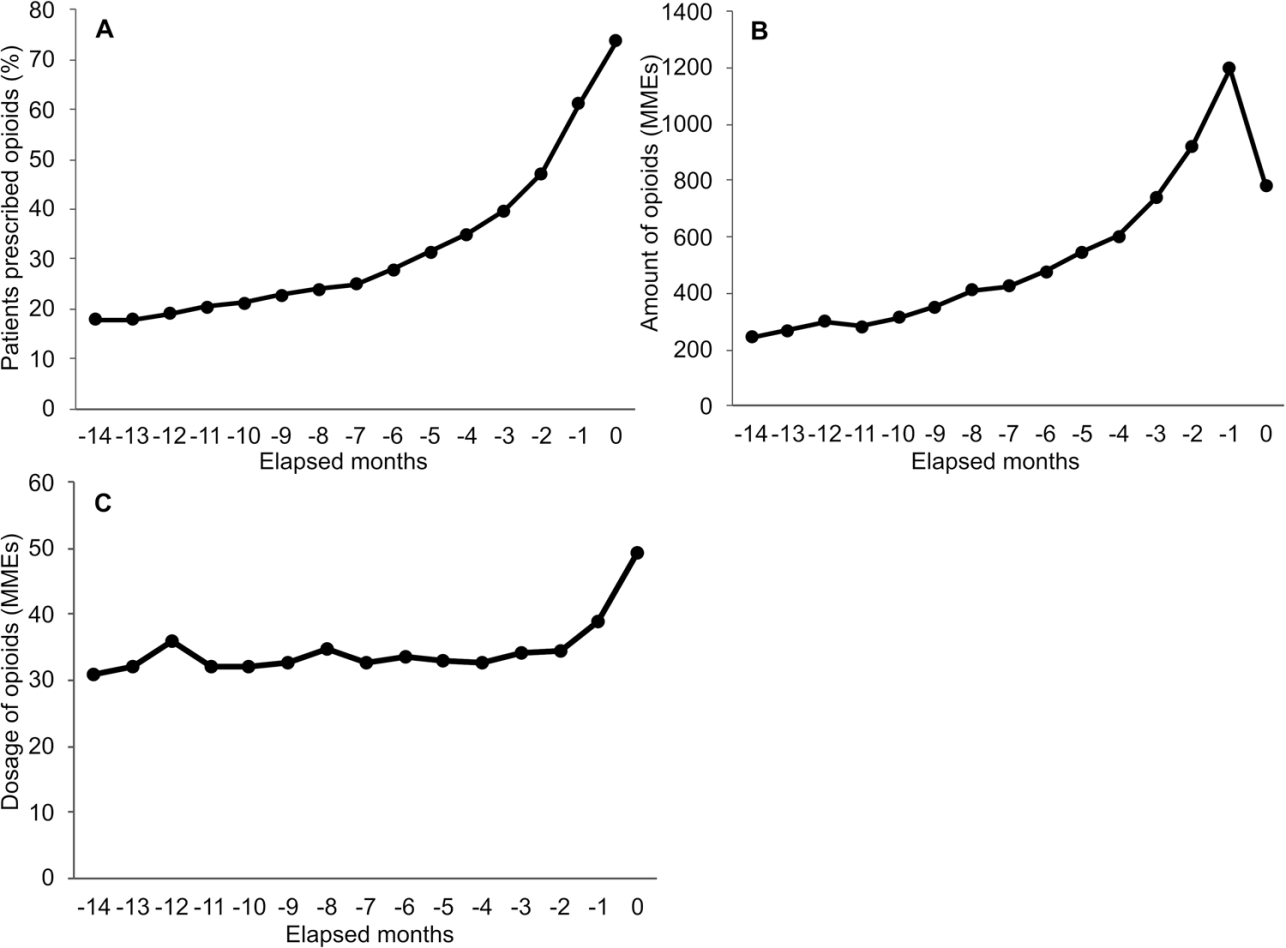
Opioid prescription status by elapsed months from death due to breast cancer. Target patients are those who died due to breast cancer.** A** Percentage of patients prescribed opioids among target patients. **B** Average amount of opioids per target patient. **C** Average opioid dosage per day. The amount and dosage of opioids are presented in morphine milligram equivalents (MMEs). MMEs, morphine milligram equivalents

The average age of patients who died due to breast cancer in the month they died was 64.5 years. The number of target patients who died due to breast cancer was 4425 (Table 3). The average (SD) period from first diagnosis to death was 18.5 (21.0) months. The percentage of target patients who were prescribed opioids increased to 73.7% of the patients in the month of death (Fig. 3A and SI Table 5). The average amount of opioids per patient and the average opioid dosage per patient per day increased as the month of death approached (Fig. 3B and 3C and SI Table 5). It should be noted that the number of days observed in the month of death for each patient depended on the date of death; the average number was likely to be 15 days. The amount of opioids prescribed during that the month depended on the number of days, so the average amount of opioids per target patient may have been underestimated.

**Table 3:** The number of target patients who died due to breast cancer and those prescribed opioids by months from death

Elapsed months	Patients^a^, No	Patients^a^ prescribed opioids, No
− 14	1988	354
− 13	2059	367
− 12	2138	407
− 11	2241	457
− 10	2334	493
− 9	2417	553
− 8	2504	599
− 7	2615	651
− 6	2719	760
− 5	2847	893
− 4	2983	1041
− 3	3169	1261
− 2	3446	1626
− 1	3952	2418
0	4425	3263

*No*, Number. ^a^Patients with breast cancer who died due to breast cancer

## Discussion

To the best of our knowledge, in this study, we examined opioid prescription status around clinical events in patients with breast cancer on a large scale for the first time in Japan. We found that the percentage of patients prescribed opioids increased in the month of surgery, after bone metastasis diagnosis and before breast cancer-related death.

The percentage of patients prescribed opioids peaked at 78% in the month of surgery. Most of these opioids may be prescribed for surgical pain and its prevention. The percentage dropped to 6.1% one month after surgery and decreased by 1–2% 3 months after the surgery in our study, which was lower than that after surgery in a previous Japanese study, 6.7% [16]. In that study, the period after surgery was defined as between surgery and chemotherapy; the gap in the results between these studies might be due to the difference in the definition of the evaluation period.

Comparing the opioid prescription after surgery to other countries, the percentage of patients prescribed opioids 3 or more months after surgery (1–2%) was lower than that 5 to 10 months after surgery (except mastectomy with reconstruction) with early-stage breast cancer, approximately 5%, in the USA [21]. One of the possible reasons for the lower percentage in our study than in the previous study may be due to the difference in the incidence of pain after surgery. In other countries, the incidence of persistent pain after breast cancer surgery was reported to be approximately 50% [25, 26]. In our study, the opioid dosage per day among patients prescribed opioids did not increase until 6 months after surgery. No increase in the dosage after surgery was shown for NSAIDs (data not shown). Accordingly, the incidence of pain after surgery seems lower in Japan than in other countries. In addition, it has been reported that the amount of opioid prescription is lower in Japan than in Western countries [27]. Nevertheless, opioids were prescribed for a certain number of patients before and after surgery, although the percentage of patients was low. Among them, the dosage of opioids gradually increased after surgery, which may suggest that the patients experienced worsened pain.

The percentage of patients prescribed opioids three to 6 months before surgery was more than 2%, which was higher than that at 3 or more months after surgery. Patients who were prescribed opioids before surgery may have had cancer pain, and some of these patients may not need opioids after the surgery. Furthermore, given an increase in the number of patients before surgery, many of these increased patients might be those who were transferred to the DPC hospitals for surgery. If many of these patients did not have pain, the increase in these patients may contribute to the decrease in the percentage of patients prescribed opioids before the surgery. As the patient group may continue to include such patients after the surgery, it may be appropriate to consider the percentage of patients prescribed opioids to be 1% as baseline before the surgery, which is the percentage 1 month before the surgery. On this assumption, the percentage after surgery was higher than that before surgery. Further research on pain status and causes for opioid prescription after surgery is needed.

Our results showing the increase in opioid use after bone metastasis suggest that bone pain increases. As breast cancer progresses, bone metastasis is frequently observed. Over 80% of patients with metastatic cancer are reported to have bone pain [28]. Considering this, increases in the percentage of patients with opioid prescriptions, the number of opioid days’ supplies, and the opioid dosage after bone metastasis seem reasonable. On the other hand, the percentage of patients with bone metastasis who were prescribed opioids after diagnosis of bone metastasis in our study was 20–30%, and the opioid dosage per day after diagnosis of bone metastasis was 20–40 MMEs, lower than that reported by a previous study from Canada and the USA; 51% of patients with breast cancer with bone metastasis were prescribed opioids in Canada [29], and 57.3% of metastatic breast cancer patients were prescribed opioids with a mean dosage of 40 − 50 MMEs one month after diagnosis of metastasis in the USA [30]. One of the reasons for this difference may be due to the restricted distribution of opioids in Japan [15]. Nevertheless, similar to that in a previous study in the USA, there was a tendency towards a decrease in the percentage of patients prescribed opioids from the month of diagnosis of bone metastasis to 2 months after the diagnosis [30]. It should be noted that patients with bone metastasis may have received other types of treatment, such as palliative radiotherapy, nerve blocks, bisphosphonate preparations, and denosumab, to reduce pain, which may have led lower amount of opioids. Differences in these treatments may be related to differences in opioid prescriptions between countries; however, we did not examine these treatments in this study.

In terms of death, we only included patients who died in DPC hospitals during hospitalization. These patients may have received much higher amounts of or more frequent opioids, resulting in an overestimation of the percentage of patients prescribed opioids and the amounts of opioids before death. Nevertheless, the percentage of patients who died due to breast cancer and were prescribed opioids before death in our study was close to that in a previous study in Italy [11]. In our study, the percentage of patients prescribed opioids and their dosage per day were approximately 40% and 34 MMEs at three months before death and approximately 70% and 50 MMEs in the month of death, respectively. In a previous study, 61.1% of breast cancer patients with very distressing pain received opioid therapy during the last 3 months before death [11]. Regarding the dosage, the International Narcotics Control Board and World Health Organization recommend an adequate dosage of opioids for patients with cancer or acquired immunodeficiency syndrome (AIDS) of 67.5 mg/day 90 days before death [31]. Compared with the recommended dosage, the average opioid dosage per day was lower in our study. The lower dosage may be due to a similar reason regarding bone metastasis described above.

This research has several limitations because of the use of data from DPC hospitals. First, this study was performed using records from a claims database, and the accuracy of the records affects the accuracy of the results. In addition, the amounts of opioids were determined based on the amounts prescribed as recorded in the database, so the amounts may have been greater than those actually taken by the patients. Additionally, opioids prescribed before the events (surgery or bone metastasis) could have been taken after the events. Second, diagnoses and medical procedures performed outside the DPC hospitals registered in the database were not recorded. Therefore, the first diagnosis or first surgery in the database may not be the first one for the patient. Third, patients who visited DPC hospitals may have severe symptoms or more complications than general patients in Japan, which may affect generalizability. Nevertheless, the influence of the difference between DPC hospital patients and general patients might be small when comparing opioid prescription status before and after events within the database. Fourth, although we performed longitudinal analyses, the number of patients included in each event year was different because each patient had a different observation period. Fifth, patients with breast cancer who had other coexisting cancers were included. These patients may have been prescribed opioids for reasons other than breast cancer. However, these opioids were also prescribed to patients with breast cancer, so we believe that these opioids should not be excluded from this study. The effect of patients with multiple cancers on the results of this study may also be similar to the actual situation. Finally, this study analyzed opioid prescription status, but the outcome of pain relief could not be analyzed because such outcome information is not available in the claims data.

## Conclusion

We illustrated opioid prescription status trends around clinical events in patients with breast cancer on a large scale in Japan. The percentage of patients who were prescribed opioids increased in the month of surgery, after the diagnosis of bone metastasis, and before death. Although the percentage of patients who were prescribed opioids was low, a certain number of patients were prescribed opioids before surgery and three months and more after surgery. Further studies on pain status and factors affecting opioid prescription are needed in patients who have undergone surgery. This study provides information that may be useful in selecting strategies to control cancer pain in patients with breast cancer.

## Supplementary information

Below is the link to the electronic supplementary material.

Supplementary file1 (DOCX 77 KB)

## Data Availability

The data that support the findings of this study are available from Medical Data Vision Co., Ltd. Restrictions apply to the availability of these data, which were used under license for this study. Data are available from the authors with the permission of Medical Data Vision Co., Ltd.
