# Supplementary material for: Opioid prescriptions at the point of surgery, bone metastasis, or death among patients with breast cancer in Japanese acute care hospitals: a claims-based, retrospective, longitudinal study
Source: Support Care Cancer. 2023 Jun 2;31(6):369. doi: 10.1007/s00520-023-07805-4 (PMC10238327; doi:10.1007/s00520-023-07805-4)

**Supplementary Information**

**SI Table 1** Definition of opioids

| Type | Generic name |
| --- | --- |
| Strong | Morphine Sulfate Hydrate |
| Strong | Morphine Hydrochloride Hydrate |
| Strong | Hydromorphone Hydrochloride |
| Strong | Oxycodone Hydrochloride Hydrate |
| Strong | Fentanyl |
| Strong | Fentanyl Citrate |
| Strong | Tapentadol Hydrochloride |
| Strong | Methadone Hydrochloride |
| Strong | Buprenorphine Hydrochloride |
| Weak | Codeine Phosphate Hydrate |
| Weak | Tramadol Hydrochloride |
| Weak | Tramadol Hydrochloride/Acetaminophen Combined Drug |
| Weak | Pentazocine |
| Weak | Pentazocine Hydrochloride |

**SI Table 2** Definition of surgery related to breast cancer

| Health claim code | Procedure name |
| --- | --- |
| 150371710 | Free skin transplantation (microscopically vascularized) (Breast reconstruction) |
| 150008910 | Free skin transplantation (microscopically vascularized) |
| 150371910 | Reconstruction surgery using tissue dilation device (breast [reconstruction surgery]) |
| 150255510 | Reconstruction surgery using tissue dilation device |
| 150120910 | Abscess of breast incision |
| 150121110 | Extirpation of breast tumor (less than 5 cm in diameter) |
| 150121210 | Extirpation of breast tumor (5 cm or more in diameter) |
| 150274610 | Segmental mastectomy |
| 150121410 | Mastectomy |
| 150413710 | Mastectomy (patients with hereditary breast and ovarian cancer syndrome) |
| 150121550 | Breast cancer cryoablation |
| 150121610 | Breast malignant tumor surgery (simple mastectomy [breast removal]) |
| 150121710 | Breast malignant tumor surgery (mastectomy and breast muscle resection ae not performed simultaneously) |
| 150121810 | Breast malignant tumor surgery (mastectomy and breast muscle resection are performed simultaneously) |
| 150121910 | Breast malignant tumor surgery (extended mastectomy [dissection performed simultaneously]) |
| 150262710 | Breast malignant tumor surgery (partial mastectomy [with axillary lymph node dissection]) |
| 150303110 | Breast malignant tumor surgery (partial mastectomy [without axillary lymph node dissection]) |
| 150316510 | Breast malignant tumor surgery (mastectomy [without axillary lymph node dissection]) |
| 150386410 | Breast malignant tumor surgery (areola preservation postmastectomy [without axillary lymph node dissection]) |
| 150386510 | Breast malignant tumor surgery (areola preservation postmastectomy [with axillary lymph node dissection]) |
| 150122150 | Breast malignant tumor surgery and bilateral axillary lymph node dissection |
| 150292210 | Mammillaplasty for inverted nipples |
| 150292310 | Reconstruction mammoplasty and mammillaplasty |
| 150316610 | Breast reconstruction using artery/muscle flap (after mastectomy) (primary) |
| 150316710 | Breast reconstruction using artery/muscle flap (after mastectomy) (secondary) |
| 150369750 | Breast reconstruction (artificial breast) (primary one-stage surgery) |
| 150369850 | Breast reconstruction (artificial breast) (primary two-stage surgery or secondary) |
| 150374010 | Breast reconstruction using gel filled artificial breast (after mastectomy) |

**SI Table 3** Opioid prescription status with first surgery related to breast cancer

The number of target patients undergoing surgery (1) and prescribed opioids at least once in each month (2), percentage of target patients prescribed opioids (3), average amount of opioids per target patient (4), average days supply per patient prescribed opioids (5), average amount of opioids per patient prescribed opioids (6) and average opioid dosage per day (7) are shown.

| Elapsed months | (1) Patients^a,^ No. | (2) Patients^a^ prescribed opioids, No. | (3) Patients prescribed opioids, % [(2)/(1)] | (4) Avg. amount of opioids per patient, MMEs | (5) Avg. days supply per patient prescribed opioids, No. | (6) Avg. amount of opioids per patient prescribed opioid, MMEs [(1)*(4)/(2)] | (7) Avg. dosage of opioid per day, MMEs [(6)/(5)] |
| --- | --- | --- | --- | --- | --- | --- | --- |
| -6 | 10,614 | 229 | 2.2% | 9.4 | 21.65 | 437.1 | 20.2 |
| -5 | 11,940 | 268 | 2.2% | 9.1 | 20.14 | 403.5 | 20.0 |
| -4 | 13,473 | 296 | 2.2% | 9.5 | 20.89 | 434.1 | 20.8 |
| -3 | 17,536 | 400 | 2.3% | 8.8 | 18.01 | 384.9 | 21.4 |
| -2 | 38,523 | 606 | 1.6% | 5.2 | 15.18 | 329.4 | 21.7 |
| -1 | 87,320 | 892 | 1.0% | 3.2 | 15.49 | 315.8 | 20.4 |
| 0 | 110,211 | 85,926 | 78.0% | 26.6 | 1.54 | 34.1 | 22.2 |
| 1 | 106,296 | 6,528 | 6.1% | 6.4 | 5.42 | 104.9 | 19.3 |
| 2 | 101,850 | 4,337 | 4.3% | 6.1 | 6.90 | 142.7 | 20.7 |
| 3 | 98,695 | 1,874 | 1.9% | 5.4 | 14.48 | 286.4 | 19.8 |
| 4 | 96,074 | 1,442 | 1.5% | 5.8 | 17.91 | 383.1 | 21.4 |
| 5 | 93,822 | 1,343 | 1.4% | 6.3 | 19.67 | 436.8 | 22.2 |
| 6 | 91,907 | 1,514 | 1.6% | 6.2 | 17.89 | 373.4 | 20.9 |
| 7 | 89,547 | 1,895 | 2.1% | 6.4 | 14.16 | 303.9 | 21.5 |
| 8 | 87,605 | 1,610 | 1.8% | 6.7 | 16.40 | 363.7 | 22.2 |
| 9 | 85,804 | 1,274 | 1.5% | 7.3 | 21.47 | 489.1 | 22.8 |
| 10 | 84,041 | 1,178 | 1.4% | 7.5 | 23.25 | 538.3 | 23.1 |
| 11 | 82,513 | 1,070 | 1.3% | 7.7 | 24.38 | 596.2 | 24.5 |
| 12 | 80,832 | 1,084 | 1.3% | 8.0 | 24.54 | 597.9 | 24.4 |
| 13 | 77,815 | 995 | 1.3% | 8.6 | 27.87 | 676.3 | 24.3 |
| 14 | 75,723 | 923 | 1.2% | 8.8 | 28.67 | 719.7 | 25.1 |
| 15 | 73,894 | 876 | 1.2% | 8.5 | 27.40 | 714.6 | 26.1 |
| 16 | 72,140 | 842 | 1.2% | 8.9 | 29.76 | 759.1 | 25.5 |
| 17 | 70,629 | 827 | 1.2% | 9.2 | 28.94 | 788.8 | 27.3 |
| 18 | 69,183 | 760 | 1.1% | 9.0 | 31.29 | 816.3 | 26.1 |
| 19 | 67,225 | 781 | 1.2% | 10.2 | 31.74 | 881.4 | 27.8 |
| 20 | 65,682 | 749 | 1.1% | 9.2 | 30.74 | 804.4 | 26.2 |

Avg. average; MMEs, morphine milligram equivalents; No, number. ^a^ Patients with breast cancer who underwent surgery

Note: the amount and dosage of opioids are presented in MMEs

**SI Table 4** Opioid prescription status with first bone metastasis diagnosis

The number of target patients diagnosed with bone metastasis (1) and prescribed opioids at least once in each month (2), percentage of target patients prescribed opioids (3), average amount of opioids per target patient (4), average days supply per patient prescribed opioids (5), average amount of opioids per patient prescribed opioids (6) and average opioid dosage per day (7) are shown.

| Elapsed months | (1) Patients^a,^ No. | (2) Patients^a^ prescribed opioids, No. | (3) Patients prescribed opioids, % [(2)/(1)] | (4) Avg. amount of opioids per patient, MMEs | (5) Avg. days supply per patient prescribed opioids, No. | (6) Avg. amount of opioids per patient prescribed opioid, MMEs [(1)*(4)/(2)] | (7) Avg. dosage of opioid per day, MMEs [(6)/(5)] |
| --- | --- | --- | --- | --- | --- | --- | --- |
| -6 | 4,085 | 235 | 5.8% | 44.0 | 30.6 | 765.4 | 25.0 |
| -5 | 4,231 | 232 | 5.5% | 51.9 | 35.3 | 946.6 | 26.8 |
| -4 | 4,396 | 285 | 6.5% | 64.5 | 36.3 | 994.9 | 27.4 |
| -3 | 4,598 | 297 | 6.5% | 70.3 | 40.5 | 1,088.9 | 26.9 |
| -2 | 4,975 | 413 | 8.3% | 88.8 | 38.4 | 1,069.8 | 27.8 |
| -1 | 6,165 | 623 | 10.1% | 99.4 | 37.7 | 983.1 | 26.1 |
| 0 | 13,146 | 3,568 | 27.1% | 203.0 | 32.6 | 748.1 | 22.9 |
| 1 | 11,301 | 3,505 | 31.0% | 371.2 | 43.2 | 1,197.0 | 27.7 |
| 2 | 9,903 | 2,464 | 24.9% | 323.0 | 44.8 | 1,298.2 | 29.0 |
| 3 | 9,111 | 1,983 | 21.8% | 316.7 | 47.8 | 1,455.3 | 30.5 |
| 4 | 8,550 | 1,738 | 20.3% | 302.8 | 47.9 | 1,489.4 | 31.1 |
| 5 | 8,085 | 1,566 | 19.4% | 285.7 | 46.1 | 1,474.8 | 32.0 |
| 6 | 7,671 | 1,420 | 18.5% | 272.2 | 45.6 | 1,470.3 | 32.3 |
| 7 | 7,305 | 1,354 | 18.5% | 267.2 | 44.1 | 1,441.4 | 32.7 |
| 8 | 6,946 | 1,246 | 17.9% | 274.6 | 45.1 | 1,530.8 | 33.9 |
| 9 | 6,642 | 1,143 | 17.2% | 269.3 | 45.8 | 1,564.7 | 34.1 |
| 10 | 6,336 | 1,053 | 16.6% | 268.1 | 45.8 | 1,613.1 | 35.2 |
| 11 | 6,091 | 986 | 16.2% | 266.8 | 46.5 | 1,648.3 | 35.5 |
| 12 | 5,821 | 909 | 15.6% | 265.9 | 45.8 | 1,702.7 | 37.2 |
| 13 | 5,541 | 861 | 15.5% | 240.1 | 44.7 | 1,544.9 | 34.6 |
| 14 | 5,309 | 806 | 15.2% | 227.0 | 44.9 | 1,494.9 | 33.3 |
| 15 | 5,111 | 784 | 15.3% | 243.3 | 44.3 | 1,586.3 | 35.8 |
| 16 | 4,898 | 732 | 14.9% | 238.3 | 45.6 | 1,594.5 | 34.9 |
| 17 | 4,676 | 684 | 14.6% | 249.3 | 47.6 | 1,704.1 | 35.8 |
| 18 | 4,521 | 663 | 14.7% | 234.6 | 45.4 | 1,599.7 | 35.3 |
| 19 | 4,311 | 631 | 14.6% | 232.6 | 45.9 | 1,589.2 | 34.6 |
| 20 | 4,130 | 592 | 14.3% | 238.5 | 48.0 | 1,663.9 | 34.7 |

Avg. average; MMEs, morphine milligram equivalents; No, number. ^a^ Patients with breast cancer who were diagnosed with bone metastasis

Note: the amount and dosage of opioids are presented in MMEs

**SI Table 5** Opioid prescription status at the time of death due to breast cancer

Among the target patients who died due to breast cancer, the average age, number of target patients who died from breast cancer (1) and were prescribed opioids at least once in each month (2), percentage of target patients prescribed opioids (3), average amount of opioids per patient who died from breast cancer (4), average days supply per patient prescribed opioids (5), average amount of opioids per patient prescribed opioids (6) and average opioid dosage per day (7) are shown.

| Elapsed months | Average age | (1) Patients^a,^ No. | (2) Patients^a^ prescribed opioids, No. | (3) Patients prescribed opioids, % [(2)/(1)] | (4) Avg. amount of opioids per patient, MMEs | (5) Avg. days supply per patient prescribed opioids, No. | (6) Avg. amount of opioids per patient prescribed opioid, MMEs [(1)*(4)/(2)] | (7) Avg. dosage of opioid per day, MMEs [(6)/(5)] |
| --- | --- | --- | --- | --- | --- | --- | --- | --- |
| -14 | 63.9 | 1,988 | 354 | 17.8% | 245.9 | 44.7 | 1,381.2 | 30.9 |
| -13 | 63.8 | 2,059 | 367 | 17.8% | 270.2 | 47.3 | 1,515.9 | 32.0 |
| -12 | 63.8 | 2,138 | 407 | 19.0% | 300.9 | 44.0 | 1,580.9 | 36.0 |
| -11 | 63.8 | 2,241 | 457 | 20.4% | 284.8 | 43.7 | 1,396.6 | 32.0 |
| -10 | 63.9 | 2,334 | 493 | 21.1% | 316.0 | 46.7 | 1,496.0 | 32.0 |
| -9 | 63.9 | 2,417 | 553 | 22.9% | 352.1 | 47.1 | 1,538.8 | 32.7 |
| -8 | 63.9 | 2,504 | 599 | 23.9% | 411.1 | 49.4 | 1,718.4 | 34.8 |
| -7 | 64.0 | 2,615 | 651 | 24.9% | 427.6 | 52.8 | 1,717.7 | 32.5 |
| -6 | 64.1 | 2,719 | 760 | 28.0% | 477.8 | 51.0 | 1,709.3 | 33.5 |
| -5 | 64.2 | 2,847 | 893 | 31.4% | 546.3 | 53.0 | 1,741.8 | 32.9 |
| -4 | 64.2 | 2,983 | 1,041 | 34.9% | 603.2 | 53.0 | 1,728.5 | 32.6 |
| -3 | 64.3 | 3,169 | 1,261 | 39.8% | 737.6 | 54.5 | 1,853.7 | 34.0 |
| -2 | 64.5 | 3,446 | 1,626 | 47.2% | 922.2 | 56.6 | 1,954.4 | 34.6 |
| -1 | 64.6 | 3,952 | 2,418 | 61.2% | 1,198.4 | 50.1 | 1,958.6 | 39.1 |
| 0 | 64.5 | 4,425 | 3,263 | 73.7% | 782.6 | 21.6 | 1,061.3 | 49.2 |

Avg. average; MMEs, morphine milligram equivalents; No, number. ^a^ Patients with breast cancer who died due to breast cancer

Note: the amount and dosage of opioids are presented in morphine milligram equivalents (MMEs)

**SI Figure 1** Patient identification

Definition of diagnosis (ICD-10 code): neoplasms, C00-D48; breast cancer, C50


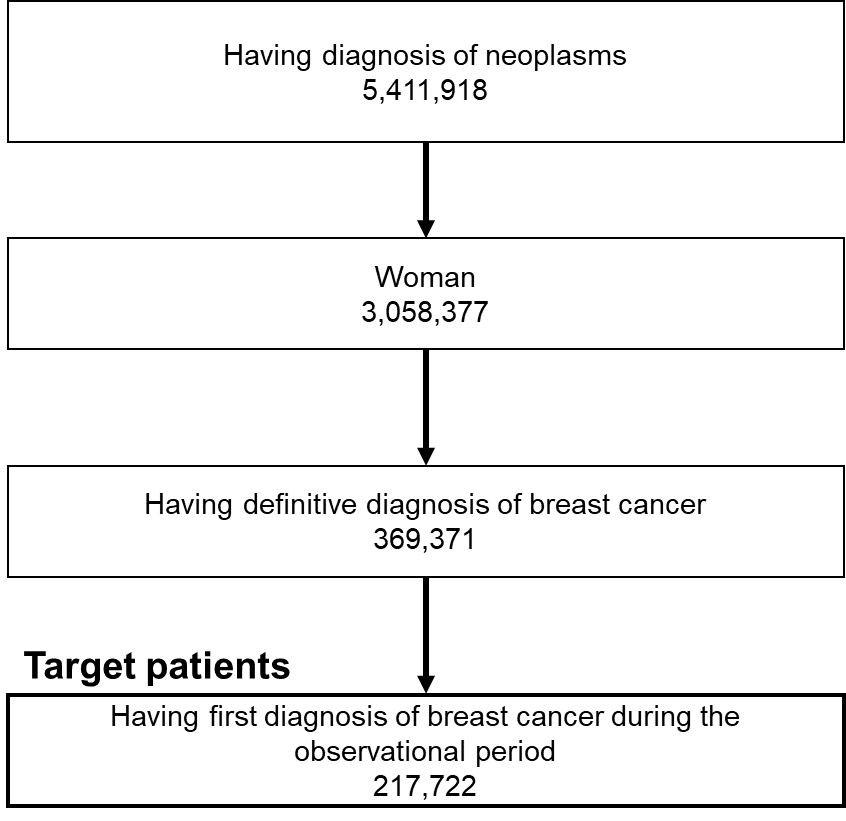

Supplement: Supplementary file 1 — Supplementary file1 (DOCX 77 KB) [file 520_2023_7805_MOESM1_ESM.docx]
